# Research at a Distance: Replicating Semantic Differentiation Effects Using Remote Data Collection With Children Participants

**DOI:** 10.3389/fpsyg.2021.697550

**Published:** 2021-08-06

**Authors:** Catarina Vales, Christine Wu, Jennifer Torrance, Heather Shannon, Sarah L. States, Anna V. Fisher

**Affiliations:** ^1^Department of Psychology, Carnegie Mellon University, Pittsburgh, PA, United States; ^2^Phipps Conservatory and Botanical Gardens, Pittsburgh, PA, United States

**Keywords:** semantic structure, semantic differentiation, semantic similarity, spatial arrangement method, semantic inference, remote data collection

## Abstract

Remote data collection procedures can strengthen developmental science by addressing current limitations to in-person data collection and helping recruit more diverse and larger samples of participants. Thus, remote data collection opens an opportunity for more equitable and more replicable developmental science. However, it remains an open question whether remote data collection procedures with children participants produce results comparable to those obtained using in-person data collection. This knowledge is critical to integrate results across studies using different data collection procedures. We developed novel web-based versions of two tasks that have been used in prior work with 4-6-year-old children and recruited children who were participating in a virtual enrichment program. We report the first successful remote replication of two key experimental effects that speak to the emergence of structured semantic representations (*N* = 52) and their role in inferential reasoning (*N* = 40). We discuss the implications of these findings for using remote data collection with children participants, for maintaining research collaborations with community settings, and for strengthening methodological practices in developmental science.

## Introduction

The field of developmental science is in urgent need of assessing remote data collection procedures. The majority of data collection in developmental science – whether observational or experimental – has traditionally relied on in-person data collection. However, there is a growing recognition that in-person data collection procedures place barriers to participation from underrepresented populations and make large samples difficult to attain. More recently, limitations to in-person data collection resulting from public health mitigation strategies due to the COVID-19 pandemic further highlighted the need for developing and evaluating remote data collection procedures. Here we replicate two semantic differentiation effects that were previously documented in 4–6-year-old children using in-person data collection and report the extent to which these effects are robust to variation in testing conditions that are typically well controlled during in-person data collection. We also describe an efficient recruitment strategy – enrolling children participating in virtual enrichment programs – that can allow researchers to broaden community partnerships. These findings point to the feasibility of conducting rapid, robust, and replicable research with children using remote data collection procedures.

### Increasing Need for Remote Data Collection With Children Participants

In the United States, developmental science has historically relied on in-person data collection procedures. At the beginning of the 20th century, a number of university-affiliated laboratories dedicated to documenting children’s development began the practice of inviting children and their caregivers to research facilities on campus to observe and assess behaviors of interest (Gesell, 1932; Ossmer, 2020). This recruitment strategy led to a number of important discoveries in the field, and is still used by many research labs to this day. However, because this approach requires participants to travel to the laboratory, it often results in study samples that are not only small (because this method is time-consuming) but also highly homogenous (because caregivers who have time and resources to travel to university laboratories come largely from White and mid- to high socioeconomic status communities). Small and homogeneous samples limit the conclusions that can be drawn from developmental studies for two reasons. First, the use of small sample sizes decreases statistical power. Statistical power is not only critical to detect true effects, but – at first glance, counterintuitively – low statistical power can *decrease* the likelihood that *significant effects* are indeed *true effects* (Button et al., 2013). In other words, the use of small sample sizes can lead to an increase of false positives. Second, homogenous samples obscure the impact of a multitude of variables on research findings, thus impeding both theoretical and empirical progress (Fernald, 2010; Henrich et al., 2010; Varga, 2011; Sugden and Moulson, 2015; Nielsen et al., 2017).

To address these concerns, researchers have developed community-based recruitment strategies that can facilitate the recruitment of larger and more diverse samples. For example, researchers have recruited and collected data in children’s museums, after-school programs, pediatricians’ offices, and mobile laboratories (e.g., Alibali and Nathan, 2010; Callanan, 2012; Cates et al., 2018). These approaches have been successful at increasing the size and diversifying study samples and are important methodological advances in the field of developmental science. However, these approaches are still limited by the geographical location of the recruitment sites and the make-up of the population they serve. For example, while recruiting participants at a children’s museum can lead to the recruitment of samples that are larger and racially more diverse, admission fees to the museum may still be a barrier to recruiting economically diverse samples.

Remote data collection procedures have the potential to help recruit larger and more representative samples of participants – in regards to race and ethnicity, income, and geographical location of the participants – into developmental studies [Scott and Schulz, 2017; Sheskin and Keil, 2018; Rhodes et al., 2020; but see Lourenco and Tasimi (2020) for how researchers should consider possible inequalities in internet access when planning remote studies]. In the last year, there was also increased interest in conducting research remotely as mitigation strategies in response to the COVID-19 pandemic severely limited the ability to collect data in person. Even with the onset of mass vaccination plans and as social distancing protocols are gradually relaxed, in-person data collection will likely not immediately return to the rates observed prior to the pandemic – making remote data collection procedures increasingly common in the coming years.

Despite the potential advantages and increased need of remote data collection procedures, and despite a number of recent studies using remote data collection with children participants (e.g., Chuey et al., 2020; Leshin et al., 2021), there is currently a gap in the *evaluation* of remote data collecting procedures used with children. It is thus critical to evaluate whether remote data collection procedures can assess constructs of interest in ways that are comparable to in-person data collection. If so, then developmental scientists can confidently use remote data collection procedures to continue to accumulate knowledge and integrate findings from remote studies with work conducted in-person.

It may seem trivial that children would perform equivalently on cognitive tasks regardless of whether they are assessed in person or remotely. Children in the United States are likely familiar with technology (Rideout, 2017; Chen and Adler, 2019), and many existing research protocols for in-person data collection are already computerized (e.g., Friend and Keplinger, 2003; Gershon et al., 2010; Fisher et al., 2013). Similarly, children are possibly more comfortable and thus more likely to engage with a task in a known setting such as their home (see Klein and Durfee, 1979; Belsky, 1980; Perry et al., 2014; Santolin et al., 2021 for related arguments). In sum, there are reasons to be optimistic about remote data collection procedures with children participants.

However, remote data collection procedures likely introduce additional variability in the setting and measurement that could limit the feasibility of remote data collection, particularly with young children. For example, while computerized assessments collected in-person standardize features such as the size of the screen used to display the task or the distance at which children sit from the screen, these factors will vary considerably when participants complete tasks remotely using their own devices. Additionally, it is also possible that children encounter more distractions when at home, that the absence of an experimenter next to the child to explain, scaffold, and redirect the child to the task when necessary, and that possible influences from caregivers would make data collection considerably less successful. Thus, it is important to ensure that – despite these potential sources of variability – data collected remotely with young children participants is comparable to data obtained from in-person assessments. While recent work has shown that remote data collection procedures can replicate the effects of lab-based studies in older children and adolescents (Nussenbaum et al., 2020), it remains an open question whether data collected remotely with young children is comparable to data obtained in-person.

Here, we address this goal by aiming to replicate two semantic differentiation effects that were previously observed in 4–6-year-old children using in-person data collection (Fisher et al., 2015; Vales et al., 2020a,b). Using remote data collection procedures, we asked whether we could conceptually replicate these effects. We did not aim to collect a representative sample or obtain a sample size larger than in prior studies (although we ultimately enrolled a larger number of participants than prior studies); rather, the main goal of this study was to provide a proof-of-concept that remote data collection procedures can measure constructs of interest in ways that are comparable to in-person data collection.

### Prior Work on Semantic Differentiation in Children

#### Measuring Semantic Differentiation Using the Spatial Arrangement Task

Organized semantic representations, linking words and the concepts to which they refer by relevant within- and across-domain distinctions, are believed to be a critical aspect of human cognition (Clark, 1973; Bjorklund and Jacobs, 1985; Gobbo and Chi, 1986). As such, there is a large interest in understanding how semantic structure develops with experience and learning, and how organized semantic representations influence other cognitive processes. Prior work suggests that children acquire structured semantic representations by exploiting the similarity structure of the entities in the world as they gradually learn about their features (Rogers and McClelland, 2004; Kemp and Tenenbaum, 2008; Hills et al., 2009). One aspect of many common domains in the world (e.g., animals, plants, clothes, tools, etc.) is that across-domain distinctions (e.g., animals vs. plants) rely on mostly non-overlapping clusters of features (e.g., only animals have eyes and can move, and only plants have leaves and roots), while within-domain distinctions (e.g., birds vs. mammals) rely on partially overlapping clusters of features (e.g., beaks and feathers vs. fur and nursing their young all overlap with the presence of eyes and mobility). This structure should lead to across-domain distinctions being generally more strongly represented earlier in development relative to within-domain distinctions.

Two recent studies directly tested this prediction using a spatial arrangement task (Goldstone, 1994) in which children were asked to arrange items by placing related items close together; the physical distance between item pairs served as a proxy for semantic relatedness, with items judged as more similar placed closer together. These studies showed that younger children (4-6 years-old) strongly differentiated items belonging to different domains – placing pairs of items of the same domain closer together relative to pairs of items of different domains (Vales et al., 2020a,b). Reliable within-domain distinctions were only visible in older children or after extended experience with a domain (Vales et al., 2020a,b).

Although prior work with adult participants has used computerized versions of the spatial arrangement method (e.g., Goldstone, 1994; Koch et al., 2020), the existing studies with children participants using this task asked children to organize physical cards on a game board (e.g., Fisher et al., 2015; Jenkins et al., 2015; Vales et al., 2020a,b). Thus, it remains an open question whether a computerized version of the spatial arrangement task would result in patterns of semantic differentiation similar to those observed in prior work. Here, we implemented and tested the first child-friendly computerized version of the spatial arrangement method.

#### Measuring Semantic Differentiation Using the Semantic Inference Task

Organized semantic representations critically support other cognitive processes, including the ability to make inductive inferences – such as assuming that members of the same within-domain group are likely to share features (e.g., Gelman and Markman, 1986; Gobbo and Chi, 1986; Coley, 2012; Fisher et al., 2015). Inductive inferences are often tested with a forced-choice semantic inference task in which children are asked to extend a property from a target item to one of a number of alternatives; for example, children might be asked whether a ‘sheep’ or a ‘cow’ shares a non-obvious feature with a ‘lamb.’ Consistent with the idea that children rely on organized semantic representations to make choices in this task and that close semantic representations compete for selection, the likelihood that children select the strongest-related item in this task is modulated not only by the similarity between the target and the match (i.e., lamb-sheep), but also by the similarity between the target and *the lure* – children are more likely to select ‘sheep’ as a match to ‘lamb’ in the presence of ‘clock’ (a lure belonging to a different domain) than in the presence of ‘cow’ (a lure belonging to the same domain) (Fisher et al., 2015).

Prior work with children using match-to-sample procedures like the one used in the semantic inference task has employed a range of number of trials (e.g., Tversky, 1985; Waxman and Namy, 1997; Fisher et al., 2015). Increasing the total number of trials completed by each participant is a crucial way to increase the precision – and thus, the power – of a task’s measurement (Forrester, 2015; DeBolt et al., 2020), but increasing the number of trials comes at the cost of possible attrition. Here, we implemented and tested a child-friendly adaptive procedure in which children could decide whether to continue or end the semantic inference task at the end of each block of trials.

### The Present Study

Together, the findings described above speak to the mechanisms supporting the acquisition of structured semantic representations and how such semantic representations support inductive inferences. The goal of this study was to conduct a conceptual replication of (1) the differences in representational strength between across- and within-domain differentiation and (2) the lure distance effect in semantic inference in 4- to 6-year-old children. If semantic structure can be assessed remotely, then one should observe similar results with a remote sample – (1) weaker representation of within-domain distinctions relative to across-domain distinctions as measured by the spatial arrangement task, and (2) lower likelihood of selecting a match in the presence of a close versus distant lure in the semantic inference task. Thus, the present study aims to conceptually replicate these two effects with remote data collection procedures.

To do so, we recruited a sample of 4- to 6-year-old children as this is the age range in which both of these experimental effects have been observed in prior work. Children participants were enrolled in an out-of-school enrichment program – aiming to provide children with hands-on, educational activities – delivered remotely by a science center. As part of the program, children completed the task on their web browser while connected in a video call with a researcher; although data collection was not fully unmoderated (cf. Rhodes et al., 2020) as caregivers were not always available during the virtual program, the tasks were set up to require minimal interaction with the researcher – all the instructions and transitions between the protocol steps were interactively delivered in the browser.

The present study also aims to extend prior work examining the relation between semantic differentiation and inductive inferences. Consistent with the idea that children rely on organized semantic representations to make inductive inferences, the degree of a child’s semantic differentiation appears to be related to their ability to make category-based inferences. Fisher et al. (2015) showed that a child’s tendency to select a within-domain category match in the inductive inference task was positively associated with how strongly the child differentiated items within a domain. Children’s within-domain semantic differentiation was assessed using the spatial arrangement method by comparing the distance at which category-matching (e.g., ‘sheep’) and habitat-matching (e.g., ‘horse’) items were placed from targets (e.g., ‘lamb’) – with larger distances indicating stronger differentiation. Children’s inductive inferences were assessed using the semantic inference task by examining the likelihood of selecting a category-matching item (e.g., ‘sheep’) as having the same property as a target item (e.g., ‘lamb’); importantly, as lure distance was not manipulated in this study, all lures in the inductive inference task were items that belonged to the same domain but not to the same category as the target (e.g., ‘frog’). In the current study we will take advantage of collecting both semantic differentiation and inductive inference assessments to further examine this relation. Specifically, we will examine the relation between within-domain semantic differentiation and the likelihood of selecting a within-domain category match in the inference task. We note that there are a number of design differences between the current study and this prior work that may make the assessment of this association not trivial; we will return to this issue when discussing the findings of this analysis.

## Materials and Methods

### Participants

We recruited a total of 58 children between 4 and 6 years of age who were enrolled in a week-long virtual enrichment program hosted by a botanical garden in Pittsburgh, PA, United States during the Summer of 2020; data were collected over three consecutive weeks, on a single day each week. To reduce economic barriers to participation, enrollment costs were partially waved. The caregiver-reported (provided to the botanical garden by 38 caregivers) gender and racial makeup of the sample was 32% male, 63% female, and 5% not reported; 79% white, 5% Black/African American, 8% Asian/Indian American, and 8% multiracial. This sample was more racially diverse than Vales et al. (2020a), which recruited from the same botanical garden but during in-person enrichment programs (see Supplementary Table S1); we will return to this point in the “Discussion” section. The same caregivers also provided their zip code information; the majority of the participants (*N* = 33) lived in Pennsylvania, with 24 unique zip codes reported; the remaining participants lived in one of four states (*N* = 4) and in Canada (*N* = 1).

Data from six children were not recorded due to technical difficulties (unstable internet connection, *N* = 4; incompatible devices, *N* = 2) and were therefore not included in the analyses reported. Forty children completed both the spatial arrangement and the inference task, and 12 children completed the spatial arrangement task but not the inference task; thus, analyses of the spatial arrangement task include 52 participants and analyses of the inference task include 40 participants.

Children completed the tasks reported here before the start or during the first day of the enrichment program activities. Because these tasks were part of the enrichment program activities, in accordance with the IRB protocol approved by Carnegie Mellon University all children enrolled in the program were invited to complete the tasks. Caregivers were given the option to have their children’s data excluded from analyses; no caregiver requested that their child’s data be excluded.

### Stimuli and Design

#### Spatial Arrangement Task

**FIGURE 1:**
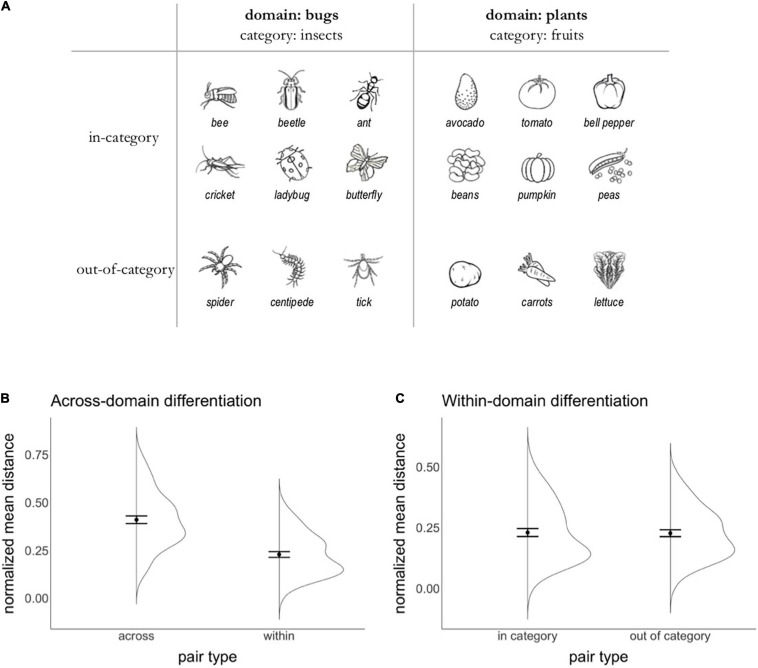
Spatial arrangement task. **(A)** Stimuli used in the Spatial arrangement task. Children were asked to arrange 18 items, half of which belonged to the domain of ‘bugs’ and the other half to the domain of ‘plants.’ To test within-domain differentiation, in-category items (i.e., insect ‘bugs’ and ‘fruit’ plants) were contrasted with out-of-category items (i.e., non-insect ‘bugs’ and non-fruit ‘plants). **(B)** Mean distance (normalized by window size) at which pairs including two items of the same domain (within) and two items of different domains (across) were placed on the screen. Error bars represent standard errors of the mean. **(C)** Mean distance (normalized by window size) at which pairs including two items of the same category within a domain (in category) and two items of different categories within a domain (out of category) were placed on the screen. Error bars represent standard errors of the mean.

The stimuli used in the Spatial Arrangement task are shown in Figure 1A and were identical to the stimuli used in Vales et al. (2020a); a comparison between Vales et al. (2020a) and the current study’s sample, task design, and results is available in Supplementary Table S1. To probe both within- and across-domain differentiation in a single trial, the stimulus set included two domains (‘bugs’ and ‘plants’) with a within-domain distinction (‘bugs’ that are insects vs. not; ‘plants’ that are fruits vs. not). Each pair of items was classified as either belonging to the same domain vs. not (i.e., whether it included any two bugs or two plants vs. one bug and one plant); this allowed us to probe across-domain differentiation. In addition, within-domain pairs were further classified as either belonging to the same within-domain group (e.g., *insect* bugs) or not (e.g., *non-insect* bugs); this allowed us to probe within-domain differentiation. Black and white line drawings representing each item were presented as individual cards with a white background against the screen’s black background.

The task was hosted in the Qualtrics platform by adapting the procedure developed by Koch et al. (2020). The pixel width and height of the center of each item was recorded, as well as the pixel width and height available on each participant’s screen; these coordinates were used to calculate the distance between all pairs of items on the screen and normalize them by each participant’s maximum possible dissimilarity (i.e., the diagonal of the participant’s screen).

#### Inference Task

Supplementary Table S2 shows all the linguistic stimuli used in the Inference task; a comparison between Fisher et al. (2015) and the current study’s sample, task design, and results is available in Supplementary Table S1. The stimulus set included six targets (all insect ‘bugs’), six matches (all insect ‘bugs’), six close lures (all non-insect ‘bugs’), six distant lures (all ‘plants’), and six novel biological properties (e.g., “vespanix cells”). To prevent children from responding based only on visually available features and to decrease overlap with the spatial arrangement task, the items in this task were not depicted and children were instead told that the items were hiding

behind trees, rocks, or grass (in blocks 1, 2, and 3, respectively) (see Fisher et al., 2015 for a similar approach).

To probe the effect of lure distance, in each block of trials each target (an insect ‘bug’) was paired with a match (another insect ‘bug’), a close lure (a non-insect ‘bug’) and a distant lure (a ‘plant’). There were a total of six targets per block, and thus a total of 12 trials per block.

Across blocks, each target was paired with a different match, lures, and property; each combination of target, match, and lures included a similar number of syllables and no overlapping word onsets. The location of the match was counterbalanced across the left and right side of the screen (with the additional constraint that the match was not presented on the same side on more than three consecutive trials), so that at the end of each block of trials the match item was equally likely to occur on either side.

There were five additional trials designed to ensure that children understood and were engaged with the task. In these trials, the target and the match items were parent/offspring animal pairs and the distant lures were vehicles (e.g., target: ‘kitty’; match: ‘cat’; lure: ‘bus’). Because the target and the match are strongly related to one another, and both are unrelated to the lure, if children understood and were engaged with the task they should reliably select the category match on these trials. Two of these trials were presented at the start of the task as familiarization trials; the other three trials were presented once in each block.

The task was hosted on the lab.js platform (Henninger et al., 2019) and embedded in Qualtrics so that the transition from the spatial arrangement task to the inference task was seamless. The participant’s response on each trial (left vs. right selection) was recorded. The files used to run these tasks are openly available: https://osf.io/67gtc/.

### Procedure

**FIGURE 2:**
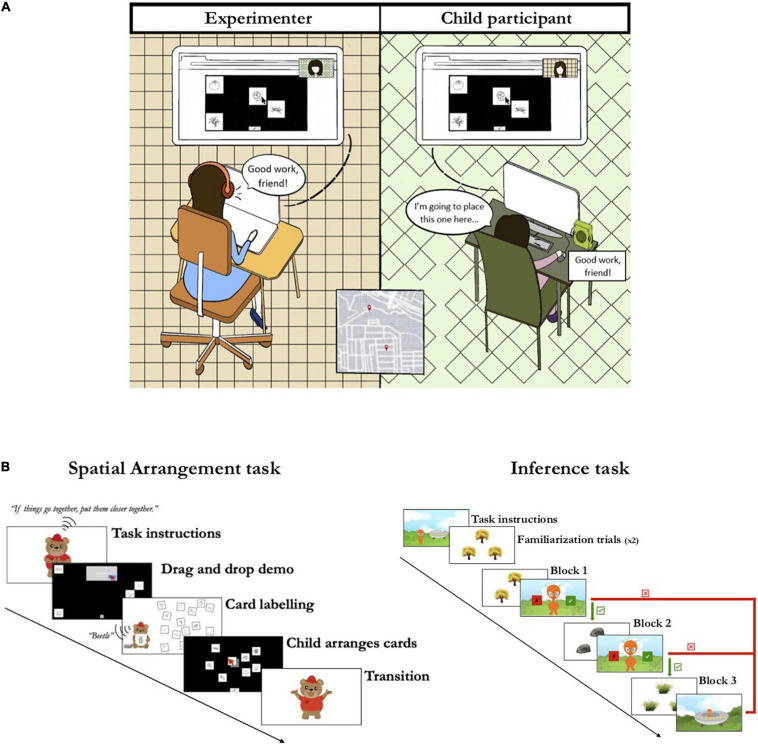
Experimental procedures. **(A)** Illustration of the experimental setup. Children were tested remotely by a trained experimenter in the Zoom platform. Children completed the tasks on a browser window; the child shared their screen so the experimenter was able to help with any technological challenges. **(B)** Sequence of events in the Spatial Arrangement task (left) and the Inference task (right); the green and right boxes and arrows represent the adaptive procedure in which children were given the option of either completing the next block of trials (green) or ending the task (red).

Children were individually tested by a trained experimenter in a breakout room in the Zoom communication platform (see Figure 2A). The experimenter started by establishing a rapport with the child; if a caregiver was present, the experimenter requested that they do not influence the child’s responses. After this initial warm-up period, the experimenter helped the child share their screen so that the experimenter could see the child’s screen and help with any experiment logistics throughout the session if needed (e.g., instructing a participant who seemed unsure how to continue); for the majority of participants no such help was needed after they started the tasks. Participants were then sent a link to the study through the Zoom messaging screen, which opened a web browser window where both tasks were completed. To ensure that the audio and video features of the browser were compatible with the study’s platform, there was a brief video that participants were asked to play.

Once the audiovisual check was performed, children started the spatial arrangement task; Figure 2B shows the sequence of events in this task. An animated video narrated by a cartoon bear explained that the goal of the game was to organize cards on the screen by placing close together cards that go together, and place far apart cards that do not. Then the video transitioned to a tutorial of how to arrange the cards on a black screen by dragging and dropping them with the mouse; four cards displaying items unrelated to the study (a bus, a duck, a duckling, and a drum) were sorted by the bear. This part of the video displayed a computer screen with a visible mouse cursor and the bear’s voice narrated while it walked through the task (e.g., “The bus does not go with the duck, so I will put them far apart”). The video ended with the bear character presenting and naming the cards that the child was asked to sort. The bear held one card at a time and labeled it (e.g., ‘beetle’); after each card was labeled, it was added to the display of already-labeled cards floating on the screen beside the bear. The cards were previewed in the same order by all children, and the labeled cards were not placed in a grid-like pattern so as to prevent biasing the child. After being shown all the cards to be sorted, children were instructed by the bear to press a button so they could start arranging their cards.

Once children advanced to the next screen, they were shown the screen where they would arrange the cards, a black background taking up the entirety of their browser window. Cards were presented one at a time in the center of the screen, in a random order for each participant. Children used their mouse, trackpad, or touchscreen to drag and drop each card anywhere on the screen. Once the first card was placed, a button at the bottom of the screen would become active and allow children to request the next card by clicking the button; this continued until all 18 cards were presented and arranged. After children arranged all cards, they were given a final opportunity to rearrange any cards before finishing the task. Upon completion, children were shown a transition video where they were thanked for their help and instructed to press a button when they were ready to start the second task.

**FIGURE 3:**
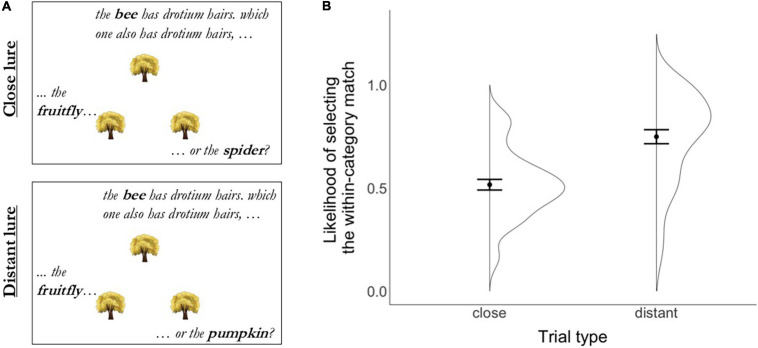
Inference task. **(A)** Illustration of the close vs. distant lure manipulation; to indicate the placement of each organism, the object referred to was synchronously jittered while being labeled. **(B)** Likelihood of selecting the within-category match in the presence of a close vs. distant lure when asked to generalize an unobservable property from a target to either the match or the lure in the Inference task. Error bars represent standard errors of the mean.

Once children advanced to the second task, a video introducing the inference task started; Figure 2B shows the sequence of events in this task. Children were introduced to an alien and told that the goal of the game was to help the alien learn about animals and plants, which were hiding. On each trial, children were shown three identical objects (trees, rocks, or a patch of tall grass) arranged in an upright triangle pattern and were told the name of the organism hiding behind each object. For example, children heard something like: “There is a bee hiding behind this tree, a fruitfly hiding behind this tree, and a spider hiding behind this tree”; each object referred to was synchronously jittered to indicate the placement of each organism. The objects on the screen were always labeled and referred to in the same order: first the object on top, then the object on the bottom left side of the screen, followed by the object on the bottom right side of the screen. After being told which organism was hiding behind each object, children were then told that the target organism had a novel biological property (e.g., “The bee has drotium hairs”) and asked to generalize this property to one of the two test organisms (e.g., “Which one also has drotium hairs?”); Figure 3A displays example trials. Children indicated their response by clicking on the item; only responses on the bottom left or right objects were accepted. Once children responded, the next trial started.

At the start of the task, after watching the introduction video, children were shown two familiarization trials that included a match and a distant lure from an unrelated domain (e.g., target: ‘kitty,’ match: ‘cat,’ lure: ‘bus’); these trials were designed to present minimal competition between the match and the lure to make sure children understood the instructions. After these familiarization trials, children were shown three consecutive blocks of trials, each consisting of 12 test trials and 1 catch trial designed in a similar manner as the familiarization trials. After each block, children were given the option of continuing to the next block or ending the task. At the end of the task, a short video showed the alien thanking the child for their help and leaving Earth on a spaceship.

Once the child completed the second task, the experimenter thanked the child and any caregivers present and answered any questions they had. The child then rejoined the group activities taking place in the enrichment program.

## Results

We examined whether we could replicate previously reported differences in representational strength between across- and within-domain differentiation (Vales et al., 2020a,b) and the lure distance effect in semantic inference (Fisher et al., 2015) using remote data collection procedures.

If an online version of the Spatial arrangement task, when delivered remotely, can provide estimates of semantic structure that are comparable to those obtained when children complete the task in person arranging physical cards on a game board, then we should see patterns of semantic differentiation similar to prior work (Vales et al., 2020a,b). Specifically, we would expect to see that children more strongly differentiate items belonging to different domains of knowledge (‘bugs’ vs. ‘plants’) relative to items within a domain (i.e., insect vs. non-insect ‘bugs’; fruit vs. non-fruit ‘plants’). To examine this prediction, we compared the average distance at which children placed pairs including items of the same vs. different domains (to examine across-domain differentiation) and pairs including items of the same vs. different categories within a domain (to examine within-domain differentiation).

Similarly, if an online version of the Inference task, when delivered remotely, can provide estimates of inferential reasoning that are comparable to those obtained when children complete the task in person, then we should see a lure distance effect similar to prior work (Fisher et al., 2015). Specifically, we would expect to see a higher likelihood of extending a property from the target object to the match in the presence of a distant relative to a close lure. To examine this prediction, we compared the likelihood of selecting the match item in the presence of a close vs. distant lure.

To examine both of these predictions, we employed a linear mixed-effects approach to test the effect of the manipulation of interest on the outcome measure. Specifically, in the *Spatial arrangement task*, we tested the effect of pair type on the raw (i.e., non-averaged per participant) Euclidean distances between pairs of items. To account for differences in the space available to arrange the cards resulting from different sizes of browser windows, these pairwise distances were normalized (i.e., divided by the by the pixel length of the diagonal of the browser window; see Koch et al., 2020 for a similar approach). To examine whether using a larger browser window influenced children’s likelihood of differentiating across or within domains, we included the size of the window in the models examining semantic differentiation. In the *Inference task* we tested the effect of lure type (close vs. distant) on the trial-by-trial likelihood of selecting the match item. Because children were given the option to continue or end the task at the end of each block, we included the number of completed blocks in the model. For each of these predictions, we provide Cohen’s *d* for the difference between the means of interest as a measure of effect size; as these predictions were tested with within-subjects manipulations, the correction suggested in Gibbons et al. (1993) was employed.

In addition to examining each task separately, we also examined the relation between the two tasks. Specifically, we examined whether the average degree of a child’s within-domain differentiation (as measured by the Spatial Arrangement task) is predictive of a child’s overall likelihood of selecting the match in the presence of the close lure in the Inference task.

Analyses were conducted in the R environment (R Core Team, 2014); except where noted we used the functions *lmer* and *glmer* from the ‘lme4’ package (Bates et al., 2015) to model continuous and binomial outcome variables, respectively. Variables were centered, with categorical variables coded using effects coding. Models were fit with the maximal random effects structure (Barr et al., 2013); we report model estimates for all models and *p*-values based on Wald tests of each model’s fixed effects. The reported effect sizes were calculated with the function *cohen.d* from the ‘effsize’ package (Torchiano, 2020). Code and data are openly available: https://osf.io/67gtc/.

### Spatial Arrangement Task

Figure 1B depicts the normalized average distance between pairs including two items from the same domain (‘within’) or from different domains (‘across’), showing that children placed pairs of items belonging to the same domain closer together relative to pairs including items from different domains. A model testing the effect of pair type (within vs. across) and window size confirmed that pair type was a significant predictor of the distance at which items were arranged on the screen [*b* = –0.18, χ^2^(1) = 45, *p* < 0.0001, Cohen’s *d* = 1.44] but window size was not [*b* = –0.000002, χ^2^ (1) = 0.002, *p* = 0.97]; the model included by-participant random intercepts and slopes for the effect of pair type. The effect size of the effect of pair type was of similar (albeit larger) magnitude relative to when data were collected in person (Vales et al., 2020a).

Figure 1C depicts the normalized average distance between pairs including two items from the same within-domain group (‘in category’) or from different groups (‘out of category’), and shows that children placed the two types of pairs at similar distances. A model testing the effect of pair type (in vs. out of category) and window size showed that neither was a significant predictor of the distance at which items were arranged on the screen [pair type: *b* = -0.003, χ^2^(1) = 0.36, *p* = 0.55, Cohen’s *d* = -0.02; window size: *b* = 0.0007, χ^2^(1) = 1.11, *p* = 0.29]; the model included by-participant random intercepts (the model including random slopes for the effect of pair type failed to converge). The effect size of the effect of pair type was of similar magnitude relative to when data were collected in person.

Together, these results provide a conceptual replication of prior work showing differences in representational strength between across- and within-domain differentiation (Vales et al., 2020a,b) using remote data collection procedures. The results also suggest that variation in the size of the web browser used to complete the spatial arrangement task is unlikely to contribute to children’s degree of differentiation when completing the spatial arrangement task; in Supplementary Material (Section C) we present additional evidence that variation in the size of the browser window is not related to the degree of semantic differentiation (see Supplementary Figure S1 in the Supplementary Material).

### Inference Task

To ensure that children understood and were engaged with the Inference task, we started by examining their performance in the familiarization and catch trials. Children were highly accurate on both the familiarization trials at the beginning of the task (*M* = 0.86, *SD* = 0.23) and the catch trials interspersed among the test trials (*M* = 0.85, *SD* = 0.23), both significantly above chance (0.5) level [familiarization: *t*(39) = 10.1, *p* < 0.0001; catch: *t*(39) = 6.96, *p* < 0.0001]. Children were also likely to complete at least two blocks of test trials (*M* = 2.25, *SD* = 0.86), further suggesting that they were engaged with the task. Because completing different numbers of trials could lead or reflect differential engagement with the task, we will include the effect of the number of blocks completed when modeling performance in the task.

Figure 3B depicts the likelihood of correctly selecting the within-category match in the Inference task across the two lure types and shows that children were more likely to select the within-category match when it was presented in the context of a distant (*M* = 0.75, *SD* = 0.22) than a close (*M* = 0.52, *SD* = 0.16) lure. A model testing the effect of lure distance (close vs. distant) and number of blocks completed on the likelihood of selecting the within-category match showed that lure distance was a significant predictor of accuracy [*b* = 1.13, *z* = 8.34, *p* < 0.0001, Cohen’s *d* = 1.22], but that the number of blocks completed did not significantly predict accuracy in the task [*b* = 0.12, *z* = 1.04, *p* = 0.28]; the model included by-participant random intercepts (the model including random slopes for the effect of lure distance failed to converge). The effect size of the lure distance manipulation was of similar (albeit larger) magnitude relative to when data were collected in person (Fisher et al., 2015).

Together, these results provide a conceptual replication of the lure distance effect reported in prior work (Fisher et al., 2015). The comparable results – both conceptually and in magnitude – across means of data collection suggest that remote data collection procedures can be used to examine semantic inferences. These results also suggest that an adaptive procedure in which children decide how many blocks of trials they complete is a viable methodological choice to maximize the number of trials collected while maintaining engagement with the task.

### Relation Between Degree of Within-Domain Differentiation and Inferences in the Presence of Close Lures

**FIGURE 4:**
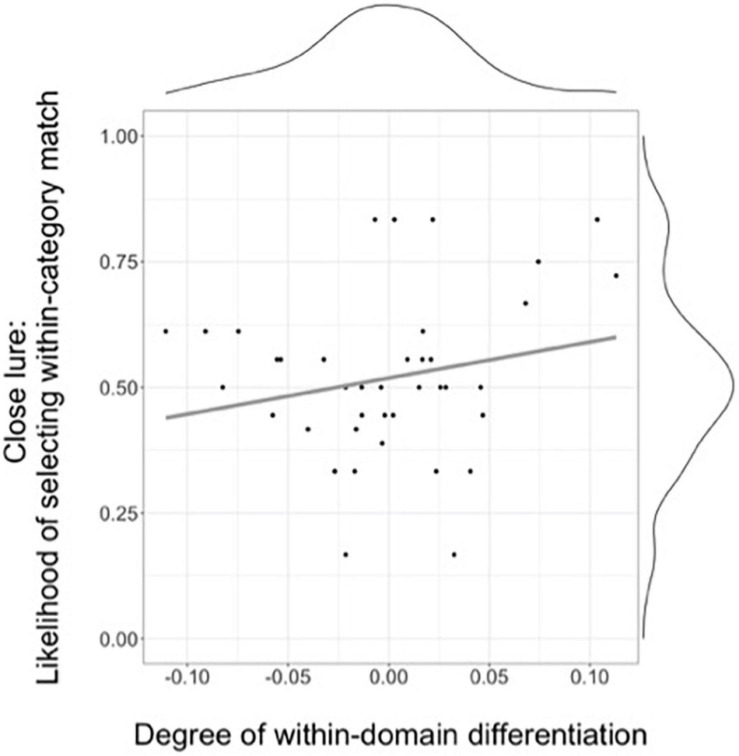
Relation between the degree of within-domain differentiation (with higher scores representing a larger distance between in-category and out-of-category pairs in the Spatial arrangement task) and the likelihood of selecting the within-category match in the close lure condition of the Inference task.

To examine the relation between a child’s within-domain semantic differentiation and the likelihood of inferring that more strongly related items within a domain are more likely to share a property, we calculated a within-domain semantic differentiation score for each child by subtracting the normalized average distance for ‘in category’ pairs from the normalized average distance for ‘out of category’ pairs’; larger difference scores thus reflect a larger degree of within-domain differentiation. Because the targets in the inference task were all insect ‘bugs,’ these difference scores only included pairs from the domain of ‘bugs.’ Figure 4 shows the association between a child’s within-domain differentiation score and their likelihood of selecting the match in the close lure condition, and suggests that there is no such relation. A linear model showed that the within-domain differentiation score was not a significant predictor of a child’s average accuracy in the close lure condition [*b* = 0.71, *R^2^* = 0.046, *F*(1,38) = 1.23, *p* = 0.19].

These results suggest that these tasks, as set up for this study, were not able to detect the association between semantic differentiation and semantic inference reported in prior work (Fisher et al., 2015). At first glance this could be taken to indicate that remote data collecting procedures are not well-suited to detect individual differences in semantic structure and/or in semantic inferences. However, it seems more likely that this lack of an association is instead due to methodological choices resulting from the main goals of this study – specifically, the goal of replicating patterns of semantic differentiation in across- versus within-domain distinctions.

As seen in Figure 4, the distribution of within-domain difference scores shows a fairly narrow range (–0.11 to 0.11) and is mostly centered around zero – suggesting that most children showed no evidence of differentiating within a domain – making it challenging to examine the role of variability in semantic differentiation. This distribution of scores stands in contrast with prior work (Fisher et al., 2015), which showed a larger range of differentiation, as well as an association between the two tasks (also see Unger and Fisher, 2019 for related evidence). The observed narrow range and distribution centered at zero is likely due, at least in part, to the fact that children in this age show fairly undifferentiated representations within a domain. However, this weak within-domain differentiation is likely exacerbated by the fact that we tested within- and across-domain differentiation *in the same trial*. We did so because this more closely replicated prior procedures (Vales et al., 2020a,b), but also to decrease the time necessary to complete the spatial arrangement task (and thus decrease possible attrition in the study) – both decisions well-aligned with the goal of replicating previously reported patterns of semantic differentiation. This, however, results in a considerable difference relative to the procedure employed by Fisher et al. (2015), who tested *triads of items* in each trial – thus providing children with a much smaller number of items at a time and thus more degrees of freedom to arrange them. In the case of the spatial arrangement task as designed for this study, the need to attend to both within- and across-domain differentiation, as well as the larger number of cards presented at once, likely reduced the likelihood of detecting individual differences in within-domain differentiation [see Experiment 2 in Vales et al. (2020b) for converging evidence]. Taken together, these results suggest that future work examining semantic structure – and in particular, individual differences in within-domain differentiation in young children – may want to consider whether to assess within-domain differentiation in separate trials and how many items to present in each trial.

## Discussion

This manuscript reports a successful conceptual replication of two semantic differentiation effects in 4- to 6–year-old children that were previously reported using in-person data collection. In the spatial arrangement task, children more strongly differentiated across domains relative to within a domain – a pattern of semantic differentiation that replicates prior work (Vales et al., 2020a,b). In the semantic inference task, children’s likelihood of selecting a within-domain category match was decreased in the presence of a close (relative to a distant) lure, replicating prior work (Fisher et al., 2015). The conceptual replication of these two effects – which speak to (1) the mechanisms by which organized semantic representations are acquired and (2) the role of organized semantic representations in supporting inferential processes – suggests that such large-sized effects can be successfully reproduced using remote data collection procedures despite the wide variation in the factors that are typically well-controlled during in-person research (such as display size and number of trials) (see also Nussenbaum et al., 2020). These results are also the first evidence that a computerized version of the spatial arrangement method can be successfully completed by children participants, and that an adaptive procedure that allows children to decide how many blocks to complete in the semantic inference task is a promising way to increase the number of trials collected from each participant while maintaining engagement with the task – both important methodological innovations, likely to be useful even in other domains of developmental science.

The use of remote data collection procedures can help strengthen developmental science. By removing a number of barriers to participation, remote data collection has the potential to increase diversity in recruited samples and facilitate the collection of larger sample sizes – both of which are critically necessary. Additionally, as a result of social-distancing measures to mitigate the spread of COVID-19, the field of developmental science is increasing the use of remote data collection procedures. The present results, showing that data collected with children participants remotely is comparable to data obtained from in-person assessments, provide a proof-of-concept that the constructs measured by these tasks can be successfully assessed remotely and thus increase the confidence that developmental scientists can continue to accumulate and integrate knowledge across different mediums of data collection.

It is important to note that the effects we set out to replicate were medium-sized; future work should evaluate if smaller-sized effects can also be replicated under the more variable testing conditions inherent to remote testing. Similarly, other tasks might be more sensitive to these more variable testing conditions; for example, increased distractions in the home environment might be more problematic in the context of experimental tasks requiring the collection of reaction time (but see Nussenbaum et al., 2020). Future work should consider these possible limiting factors when planning online data collection. We also note that not all children completed both tasks, with about 20% of children who completed the spatial arrangement task not completing the inference task. Prior work examining the relation between these two tasks (Fisher et al., 2015) conducted the two tasks in two separate sessions, as the study included numerous measures at multiple time points. As such, we do not know whether the attrition rate observed here would be similar to in-person data collection procedures. Future work intending to collect multiple measures per participant within the same study session should consider the attrition rate observed here and decide whether conducting multiple sessions may be a better approach to their goals.

Remote data collection procedures by themselves will not be sufficient to realize the promise of increasing diversity in study samples. The sample in this study was a convenience sample resulting from an ongoing partnership with the science outreach team at a local botanical garden, and thus we did not aim to obtain a geographically diverse sample (although some families joined from out-of-state, which would have been unlikely had the programs taken place in person). When planning this collaboration, we took steps to increase diversity in the demographics of children participants, both through publicizing the camps in underserved neighborhoods and by reducing enrollment costs – and these efforts seem to have been successful to some extent, as we saw an increase in non-white participants relative to a prior collaboration (Vales et al., 2020a) and considerable variability in the neighborhoods (i.e., zip codes) where the participants lived. However, because these camps were moved to a remote medium as a result of social-distancing guidelines due to the COVID-19 pandemic in the Spring and Summer of 2020, there were considerable changes in enrollment as family and childcare circumstances quickly changed. This makes it difficult to know whether our efforts to broaden participation could have been more successful under different circumstances. Indeed, as Lourenco and Tasimi (2020) note, researchers must continue to take steps to ensure equitable access for families from disadvantaged backgrounds, especially during a pandemic when access to internet might be even more challenging (e.g., libraries might not be open to the public).

The current study failed to find an association between the degree of a child’s within-domain differentiation and their likelihood of selecting the matching within-domain item in the presence of a close (i.e., belonging to the same-domain) lure. Although this could be taken to indicate that remote data collection procedures are not well-suited to detect individual differences in these two processes, it seems more likely that the lack of an association between the two tasks is instead due to the limited range of scores and a distribution centered around zero that was observed for the within-domain difference scores. We believe these undifferentiated scores are a result of both weak within-domain differentiation (consistent with the patterns found in the spatial arrangement task) and the fact that both within- and across-domain differentiation were tested *in the same trial*, which reduced the degrees of freedom for arranging individual cards. This is a crucial difference relative to prior work (Fisher et al., 2015), and in requiring children to simultaneously attend to both distinctions might have reduced the odds that children noticed within domain distinctions. Prior work using this task suggests that these are important methodological considerations (Vales et al., 2020b), and we believe future work intending to use remote assessments of semantic structure and semantic inferences should consider the goals of the assessments when deciding whether to examine within- and across-domain differentiation in the same or separate trials.

The recruitment strategy we used – recruiting children participating in a virtual enrichment program – can also be a useful tool for researchers to maintain and extend their partnerships with community settings during the current limitations to in-person testing. Over the course of only three weeks, with a single 2.5 h-long session involving 5–7 researchers each week, we recruited and tested more than 50 children. The researcher involvement was fairly minimal, and it is likely that with some improvements to the usability of the tasks it would be possible for children to complete these tasks without any researcher involvement. Partnerships between basic science researchers and educators are an important component of developmental science and can be mutually beneficial for the researchers and the educators (Osberg, 1998; Callanan, 2012; Haden, 2020; Mulvey et al., 2020). The COVID-19 pandemic has propelled the development of virtual learning programs (Bell, 2020); this study illustrates how researchers can leverage this reality to maintain existing partnerships within their local communities and possibly develop new ones with science centers that were previously geographically inaccessible – and in so doing, study developmental change in ecologically valid settings (Golinkoff et al., 2017).

In sum, the current results suggest that the spatial arrangement task and the semantic inference task can be successfully employed to remotely assess semantic structure. This allows future work using these tasks to be aggregated with prior work using in-person data collection procedures. This also provides researchers with alternative ways to recruit larger and more diverse samples, and thus continue to strengthen practices in developmental science.

## Data Availability Statement

The datasets presented in this study can be found in online repositories. The names of the repository/repositories and accession number(s) can be found below: https://osf.io/67gtc/.

## Ethics Statement

The studies involving human participants were reviewed and approved by Carnegie Mellon University Institutional Review Board. Written informed consent from the participants’ legal guardian/next of kin was not required to participate in this study in accordance with the national legislation and the institutional requirements.

## Author Contributions

CV, CW, and AF designed the study. CV and CW processed the data and performed the statistical analyses. CV wrote the first version of the manuscript. CW wrote sections of the manuscript. All the authors contributed to manuscript revision, read, and approved the submitted version.

## Conflict of Interest

The authors declare that the research was conducted in the absence of any commercial or financial relationships that could be construed as a potential conflict of interest.

## Publisher’s Note

All claims expressed in this article are solely those of the authors and do not necessarily represent those of their affiliated organizations, or those of the publisher, the editors and the reviewers. Any product that may be evaluated in this article, or claim that may be made by its manufacturer, is not guaranteed or endorsed by the publisher.
