# Supplementary material for: Research at a Distance: Replicating Semantic Differentiation Effects Using Remote Data Collection With Children Participants
Source: Front Psychol. 2021 Aug 6;12:697550. doi: 10.3389/fpsyg.2021.697550 (PMC8377201; doi:10.3389/fpsyg.2021.697550)
Supplement: Supplementary file 1 [file Data_Sheet_1.pdf]

SUPPLEMENTAL MATERIALS

<u>(A) Comparison of results from in-person and remote data collection using the Spatial Arrangement and Inference tasks</u>

Table S1 – Description of the two tasks used in the current study (remote data collection) and in prior work (in-person data collection).

		**Vales, States, & Fisher (2020)**	**Fisher, Godwin, & Matlen (2015; Exp. 1)**	**Current study**
**Spatial Arrangement task**	**Sample**	29 children; age: M = 4.5 years, SD = 0.6.<br>Recruited from a group enrolled in an enrichment program in an urban area in the northeastern of the United States.<br>90% white, 7% Asian/Indian American, 3% not reported.<br>Tested in person.		52 children between 4 and 6 years old.<br>Recruited from a virtual enrichment program.<br>79% white, 5% Black/African American, 8% Asian/Indian American, and 8% multiracial (due to the nature of the IRB protocol, demographic information was only available for the entire sample).<br>Tested remotely.
	**Task description**	Children were asked to sort cards depicting individual items on a game board such that items that go together were placed closer together. The cards were shown and labeled to the child before sorting. Children could rearrange the cards at any point during the session.		Children were asked to sort cards depicting individual items on their computer such that items that go together were placed closer together. The cards were shown and labeled to the child before sorting. Children could rearrange the cards at any point during the session.
	**Design**	The items tested belonged to one of two domains ('bugs' and 'plants'), and to one of two within-domain groups (insects vs. non-insect 'bugs', and fruits vs. non-fruit 'plants'). Children sorted a total of 18 cards in a single trial. Children were tested on the first (pretest) and last (posttest) day of the enrichment program.		The items tested belonged to one of two domains ('bugs' and 'plants'), and to one of two within-domain groups (insects vs. non-insect 'bugs', and fruits vs. non-fruit 'plants'). Children sorted a total of 18 cards in a single trial. The stimuli were identical to those used in Vales, States, & Fisher (2020); see Figure 1A for the full stimulus set.

	**Main results**	At both pre- and posttest, children placed pairs including items of the same domain closer together relative to pairs including items of different domains (pretest: Cohen's *d* = 0.55). Only at posttest did children place pairs including two items of the same within-domain group closer together relative to pairs including items of different within-domain groups (pretest: Cohen's *d* = -0.05).		Children placed pairs including items of the same domain closer together relative to pairs including items of different domains (Cohen's *d* = 1.44). Children did not place pairs including two items of the same within-domain group closer together relative to pairs including items of different within-domain groups (Cohen's *d* = -0.02).
**Inference task**	**Sample**		15 children; age: *M* = 4.41, *SD* = 0.27. Recruited from a laboratory school of a private university in the United States. Predominantly from middle to high socio-economic status households. Tested in-person.	40 children between 4 and 6 years old. Recruited from a virtual enrichment program. 79% white, 5% Black/African American, 8% Asian/Indian American, and 8% multiracial (due to the nature of the IRB protocol, demographic information was only available for the entire sample). Tested remotely.
	**Task description**		Children were told that a familiar item had a novel property (e.g., "this *rat* has *omat* inside") and asked which of two other familiar items (e.g., *mouse* or *skunk*) also had that property. The items were presented as hiding behind closed doors; items and properties were presented verbally by the experimenter. Children indicated their choice by pointing to the door.	Children were told that a familiar item had a novel property (e.g., "this *ladybug* has *muscanic pores*") and asked which of two other familiar items (e.g., *firefly* or *centipide*) also had that property. The items were presented as hiding behind trees, rocks, and tall grass (in blocks 1-3, respectively); items and properties were presented auditorily in the browser window. Children indicated their choice by clicking on the tree/rock/grass.
	**Design**		Manipulation: each target item (e.g., *rat*) was paired with a same-category match (e.g., *mouse*); across trials, each target-match pair was presented with either a close (e.g., *skunk*) or distant (e.g., *spoon*) lure. Number of trials: children completed a total of 10 trials, half in each lure condition.	Manipulation: each target item (e.g., *ladybug*) was paired with a within-category match (e.g., *firefly*); across trials, each target-match pair was presented with either a close (e.g., *centipede*) or distant (e.g., *tomato*) lure. Number of trials: children completed up to 3 blocks of trials; each block had 12 trials, half in each lure condition. The stimuli were conceptually similar but not identical to Fisher, Godwin, & Matlen (2015); see Table A2 for the full stimulus set.
	**Main results**		Children's likelihood of selecting the match in the presence of the distant lure (*M* = 0.75, *SD* = 0.29) was higher relative to the close lure (*M* = 0.58, *SD* = 0.30; Cohen's *d* = 0.58)	Children's likelihood of selecting the match in the presence of the distant lure (*M* = 0.75, *SD* = 0.22) was higher relative to the close lure (*M*=0.52, *SD*=0.16; Cohen's *d* = 1.22)

<u>(B) Linguistic stimuli used in the Inference task</u>

Table S2 – Complete list of linguistic stimuli used in the Inference task. The close vs. distant lure manipulation was implemented across trials. The target, match, and lure were presented hiding behind trees (block 1), rocks (block 2), and tall grass (block 3). At the end of each block children were given the option to continue or end the task.

Target	Match	Close Lure	Distant Lure	Property
		Block 1		
Ant	Flea	Tick	Bean	*Vespanix* cells
Butterfly	Praying Mantis	Roly Poly	Bell Pepper	*Formical* mucus
Beetle	Mosquito	Tarantula	Potato	*Drotium* hairs
Ladybug	Firefly	Centipede	Tomato	*Muscanic* pores
Bee	Fruitfly	Spider	Pumpkin	*Tescium* nerves
Cricket	Grasshopper	Millipede	Avocado	*Plaxium* blood
		Block 2		
Ant	Praying Mantis	Roly Poly	Bell Pepper	*Formical* mucus
Butterfly	Grasshopper	Millipede	Avocado	*Plaxium* blood
Beetle	Fruitfly	Spider	Pumpkin	*Tescium* nerves
Ladybug	Flea	Tick	Bean	*Vespanix* cells
Bee	Mosquito	Tarantula	Potato	*Drotium* hairs
Cricket	Firefly	Centipede	Tomato	*Muscanic* pores
		Block 3		
Ant	Fruitfly	Spider	Pumpkin	*Tescium* nerves
Butterfly	Flea	Tick	Bean	*Vespanix* cells
Beetle	Grasshopper	Millipede	Avocado	*Plaxium* blood
Ladybug	Mosquito	Tarantula	Potato	*Drotium* hairs
Bee	Firefly	Centipede	Tomato	*Muscanic* pores
Cricket	Praying Mantis	Roly Poly	Bell Pepper	*Formical* mucus

(C) Supplemental analysis: Is the size of the browser window related to the degree of semantic differentiation?

We additionally examined whether variation in the size of the browser window used to complete the spatial arrangement task was related to the degree to which children differentiated across or within domains. One possibility is that using a smaller window makes it more challenging for participants to differentiate items. We note that the relative size of the cards is preserved across varying sizes of browser windows; nevertheless, it is possible that windows (and cards) perceived as smaller make participants less likely to differentiate items. If that were the case, then we should expect to see a larger degree of differentiation for larger window sizes.

To calculate a participant's *degree of across-domain differentiation* we subtracted the normalized average distance for 'within' pairs from the normalized average distance for 'across' pairs; larger difference scores thus reflect a larger degree of across-domain differentiation. Similarly, to calculate a participant's *degree of within-domain differentiation* we subtracted the normalized average distance for 'in category' pairs from the normalized average distance for 'out of category' pairs'; larger difference scores reflect a larger degree of within-domain differentiation. Figure S1 depicts the relation between a participant's window size and their degree of across- and within-domain differentiation. It does not seem to be the case that using a larger screen increases the odds that children better differentiate across domains or within a domain. A model testing the effect of window size (centered) and score (across- vs. within-domain) on the degree of differentiation showed that window size was not a significant predictor of degree of differentiation [b=-0.00009, χ^2 (1)=1.93, p=0.17]; the type of score (across- vs. within-domain) was a significant predictor of the degree of differentiation [b=-0.018, χ^2 (1)=51.12, p<0.0001], in agreement with the distance analyses reported in the manuscript. There was no interaction between the two predictors [b=-0.0002, χ^2 (1)=2.85, p=0.09]. Together, these results converge with those presented in the manuscript and suggest that variation in the size of the browser window used to complete the spatial arrangement task is unlikely to determine a participant's degree of differentiation in the task.

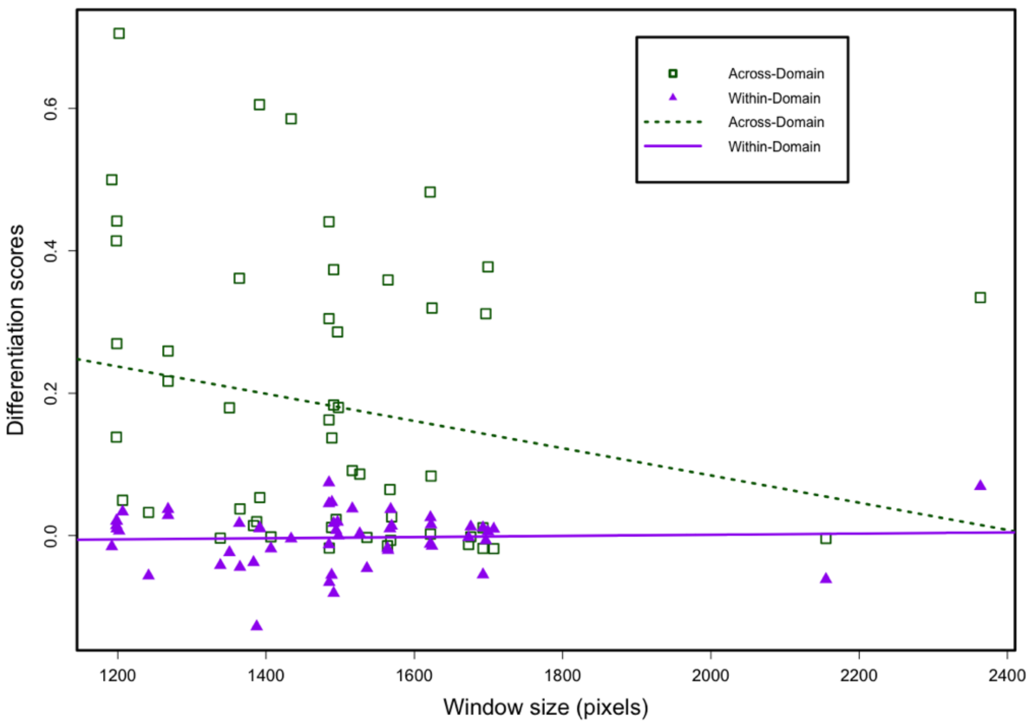

Figure S1. Relation between the diagonal of the browser window (in pixels) used to complete the spatial arrangement task and the degree to which children differentiated within and across domains. Each point represents the corresponding differentiation score for each participant; the lines show the best fitting line for a regression model. Although the type of score (within vs across) was a significant predictor of differentiation scores, the size of the window was not.
